# Multiplexed Ultra-Sensitive Detection of Cr(III) and Cr(VI) Ion by FET Sensor Array in a Liquid Medium

**DOI:** 10.3390/s19091969

**Published:** 2019-04-26

**Authors:** Suman Shahim, Revathi Sukesan, Indu Sarangadharan, Yu-Lin Wang

**Affiliations:** 1 Institute of Nano Engineering and Microsystems, National Tsing Hua University, Hsinchu 300, Taiwan; s106035421@m106.nthu.edu.tw (S.S.); s105035422@m105.nthu.edu.tw (R.S.); indu@gapp.nthu.edu.tw (I.S.); 2 Department of Power Mechanical Engineering, National Tsing Hua University, Hsinchu 300, Taiwan

**Keywords:** chromium, extended gate field-effect transistors (FETs), ion-selective membrane (ISM), sensor array, multiplexed heavy metal ion detection, lab-on-a-chip

## Abstract

Chromium, one of the top five toxic heavy metals ranked according to significance in public health by WHO, exists as Cr(III) which is naturally occurring or Cr(VI) which is anthropogenic in origin. The EPA specifies the maximum contaminant level in drinking water to be 10^−6^ M or 0.1 mg/L or 100 ppb for the total dissolved Cr. To ensure the water consumed by the population has these pollutants below the safe threshold, this report demonstrates a field effect transistor (FET) based sensor design incorporating a highly target specific ion-selective membrane combined with extended gate technology which manifests sensitivity exceeding the Nernst limit aided by the high field effect in the short gap region of extended gate technology. Characterization and repeated testing of the portable device revealed a commendable calibration sensitivity of 99 mV/log [Cr^3+^] and 71 mV/log [Cr^6+^] for Cr(III) and Cr(VI) respectively, well surpassing the Nernst limits of sensitivity and offering a detection limit lower than ion-selective electrodes (10^−6^ M), and comparable to the expensive benchtop laboratory instrument, ICP-MS. This report presents a robust, easy to fabricate, economic and efficient handheld biosensor to detect the chromium in a liquid sample whether it exists as Cr(III) or Cr(VI).

## 1. Introduction

Heavy metal contamination, leading to crippling effects of bio-accumulation, is an issue demanding utmost attention today as it impacts human health and environment [1]. Among the various germane threats to human health in the present era, heavy metals serve as an elusive adversary. This can be attributed to their ubiquity, toxicity at trace levels and bio-accumulation of these toxic elements in an organism over time which manifests in carcinogenic effects, multiple organ failure and so on [2,3]. Exposure to these detrimental toxicants may be varied; sources of natural origin such as lithogenic weathering, or anthropogenic such as industrial effluent and occupational hazards [4]. Among the most toxic of these species called heavy metals are arsenic, cadmium, chromium, mercury, and lead [5].

Anthropogenic industrial and manufacturing processes contribute to surging proportions of heavy metal in the lithosphere, hydrosphere, and atmosphere. The anomalous degradation of water quality by effluent discharges raises issues of significant global health concern. Chromium exists as the 21st most abundant element on the earth’s crust and is substantially used in leather tanning, electroplating, steel manufacturing, pigment, and alloy manufacturing industries. First discovered in 1797 by Louis Nicholas Vanquelin, it was named after the Greek word ‘chroma’ which meant color. The ruby, sapphire, and emerald derive their color from small amounts of chromium [6]. The industrial discharges released contaminate water with Cr(VI) and Cr(III) ultimately causing genotoxicity to flora and fauna. Hence, dynamic water quality monitoring by detecting the heavy metal presence in tap water, canned liquid food items, etc. will be of profound application if the sensor design is reasonably priced and operated with a simple user interface. Cr(III) and Cr(VI) has been identified as group III and group I carcinogen respectively by the United States Environment Protection Agency and International Agency for Research on Cancer [2,7,8]. In plants, heavy metals entering the tissue release reactive oxide species (ROS), causing lipid membrane damage leading to impaired functioning of chlorophyll and photosynthetic activity stunting growth. In higher organisms, heavy metals such as Cr(VI) react with biological reductants such as thiols and ascorbate resulting in mutagenic effects altering the DNA and ultimately leading to cancer [9]. Tanneries release large quantities of untreated chromium into water bodies. Leaching of soils near industrial areas also increases the concentration of this carcinogen in the hydrosphere [10]. Chromium exists in many oxidation forms but the most stable is Cr (0) and Cr(III). Higher states are highly reactive, good oxidizing agents and readily get reduced after release into the environment to lower states [11]. Groundwater contamination of Hinkley, California by carcinogen Cr(VI) owing to the dumping of 370 million gallons of chromium-tainted wastewater plume by Pacific Gas and Electric back 1960 is still causing prostate, cervical, breast and stomach cancers and respiratory problems in the demographic population [12,13]. Hence, it is essential to monitor the concentration of Cr(III) and Cr(VI) in drinking water to safeguard the health of the population.

The US Environmental Protection Agency has established the permissible limits of total chromium at 0.1 mg/L or 100 parts per billion (10^−6^ M). Maximum contaminant level (MCL) covers both III and VI oxidation forms as they are interconvertible in the human body [14]. In stainless steel welding, wood preserving, and other pigment and electroplating units, the workforce is exposed to chromium as an occupational hazard. The Occupational Safety and Health Administration, United States Department of Labor has limited air-borne exposure (processes releasing dust, mists, fumes) to below concentrations at or above 0.5 µg/m^3^ as an 8-h time-weighted average (TWA) [15]. Existing conventional techniques of detection or measurement of concentration comprise atomic absorption spectrometry (AAS), atomic emission spectrometry (AES), X-ray spectroscopy, inductively coupled plasma mass spectrometry (ICP-MS), high-pressure liquid chromatography (HPLC). They suffer from drawbacks such as complex procedure, the requirement of trained personnel, are expensive, time-consuming and require a laboratory setup though they offer considerably low detection limits [16,17,18,19,20]. Electrochemical methods though less expensive have higher detection limits than the maximum safe limits [21,22,23,24].

In this study, we develop a portable, robust ion-selective membrane (ISM) coated, extended gate field effect transistor (ISMFET) which gives increasing response of drain current with an increase in the concentration of chromium ions in the dropped test solution. Extrapolating the calibration curve can determine the concentration of the target and helps to monitor drinking water quality. The detection limit is lower than safe limits set by WHO and the compact device with nearly ten minutes response time has an operating life of a few months and sensitivity irrespective of pH from 3–8. The detection method employed for Cr(III) and Cr(VI) differ only by one reagent; the target specific ionophore that selectively binds with the target. The membrane was experimentally determined to have commendable selectivity; higher affinity towards chromium and no response to other interfering cations. By combining ion-selective membrane with an extended gate device connected to a FET, and introducing the electric double layer structure with an extremely small gap distance enables us to attain a sensitivity higher than the Nernst response, and also improves the detection limit to 10^−11^ M for Cr(III) and Cr(VI). The sensor having a simple user interface, low detection limits, cost efficiency with short response time is ideal and convenient for water quality monitoring use by common man to safeguard his health from the adverse effects of chromium ingestion.

## 2. Materials and Methods

**Figure 1:**
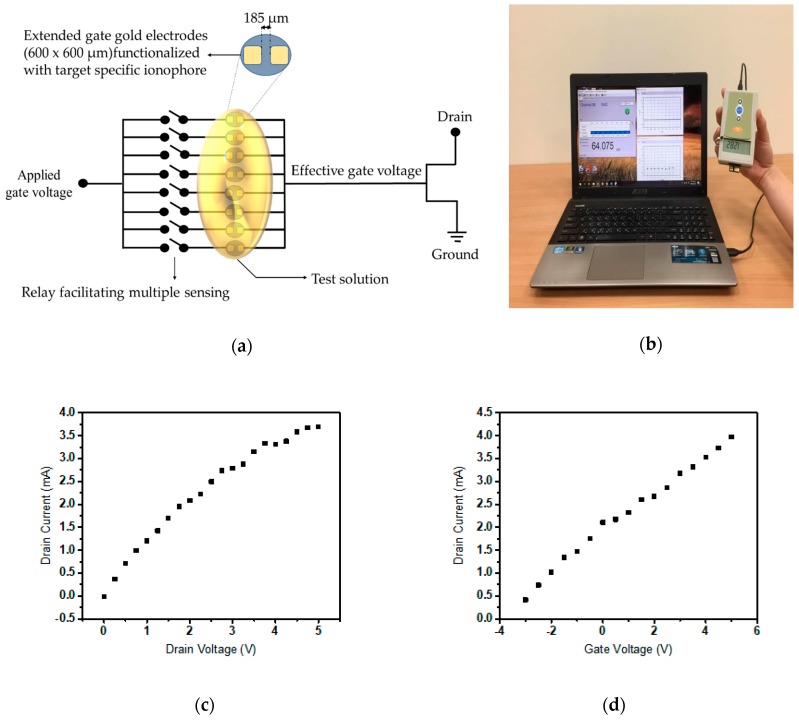

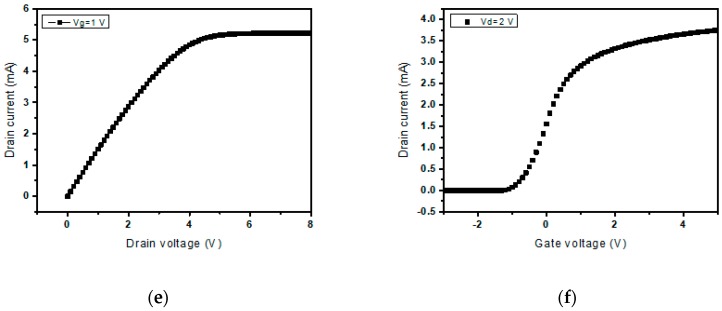
(**a**) Schematic representation of circuit implemented as sensor detection system; (**b**) Real world model of the device implanted; (**c**) plot of drain current to the voltage across the drain-source terminal; (**d**) graph of output drain current to the input gate voltage of the extended gate field effect transistor (FET) sensor; (**e**) output characteristics of LND 150; (**f**) transfer characteristics of LND 150.

A diagrammatic depiction of the ISM coated FET based sensor array is displayed in Figure 1a portraying the multiple sensing elements; the two electrode extended gate system array connected to the gate terminal of the FET which operates as a transducer. The gate terminal of the n-channel depletion-mode DMOS FET (#LND150) is extended and ends in a pair of sensing electrodes covered with PVC matrix Cr selective sensing material. The ionophore which binds with Cr(III) or Cr(VI) belongs to a category of supramolecular receptors exhibiting cavities that match the size of the target and corresponding charge distribution. The sensing element constitutes the 600 × 600 µm^2^ open area separated by 185 µm which is immobilized with the target-selective membrane composition exposed by photolithography to remove 2 µm thickness SU-8 photoresist. A single sensing unit comprises a gold electrode pair; a sensing electrode the relayed to the MOSFET gate terminal which is responsible for conducting the capacitance modulated voltage to FET and other; the reference electrode connected to the applied gate voltage. At the reference electrode, a short time pulsed gate voltage with an amplitude of 1 V and width 100 µs is administrated as the gate bias. A drain-source voltage of 2 V is applied throughout the process. The overall portable and user-friendly system used for measurement is shown in Figure 1a,b, where the extended gate chip is inserted through a PCI-E port to the micro-controller or prototype handheld unit which is interfaced to the personal computer for visual representation and data collection. Device characteristics of the portable sensor unit comprising the FET and extended gate electrodes is depicted in Figure 1c,d using the output characteristics and transfer characteristics. Figure 1e,f show the output and transfer characteristics of the FET. The gold electrodes are fabricated by metal deposition over thermo-curable epoxy resin poured into polydimethylsiloxane (PDMS) molds kept in the oven at 1.5 h at 165 degree Celsius. Electron beam evaporator is used to deposit Ti (200 Å) and Au (2000 Å) on the epoxy substrate acceded by lift-off of photoresist used for patterning.

**Figure 2:**
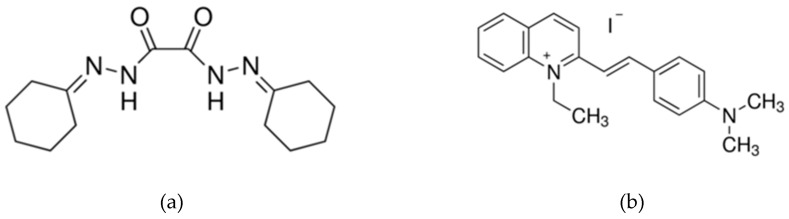
(**a**) chemical structure of Bis(cyclohexanone)oxaldihydrazone, the Cr(III) specific ionophore; (**b**) Cr(VI) selective compound quinaldine red.

For the development of ion-selective membrane, high molecular weight PVC, plasticizer 2-nitro phenyl octyl ether, anion-excluder sodium tetra phenyl borate, Cr(III) selective ionophore bis(cyclohexanone)oxaldihydrazone (Figure 2a), and Cr(VI) specific ionophore quinaldine red (Figure 2b) were purchased from Sigma Aldrich and used without any further treatment. Chromium nitrate and potassium chromate were used as salts of Cr(III) and Cr(VI) to spike in 0.02X phosphate buffer saline standard solution. The membrane immobilized on the sensing element is composed by thoroughly dissolving 114 mg of PVC, 150 mg plasticizer, 6 mg NaTPB and 30 mg of the corresponding ionophore in 3 mL of organic polar solvent THF. Sonication and magnetic stirring were used to ensure uniform composition through the volume. The semi-liquid is carefully dropped on the gate sensing electrode using a pipette and left to dry for 24 h. The medium in which successive spiked concentrations of the target ion was phosphate buffer saline (PBS) at a concentration of 0.02X ionic strength. Once the characterization or calibration curve of the sensor is determined in the standard buffer solution, the device is used for testing in river water. 0.25 µL of the composition is carefully pipetted onto the gate sensing region encapsulating both the target and reference electrode. Half the sensors in the array or extended gate chip are immobilized with membrane composition specific to Cr(VI) and others with Cr(III) selective drops to enable multiplexed detection of both ions and ultimately to use the calibrated sensor to measure total chromium concentration in the test solution. River water, canned foods, blood or any liquid buffer could be used to prepare the solution under detection test. The immobilization of supramolecular receptor is rendered complete after 24 h to ensure the solvent completely vaporizes, leaving the PVC matrix behind ready for calibration.

## 3. Results and Discussion

The measurement of electrical properties of the device is expressed by the quantity ‘gain’; current in microampere range which is the difference in drain current of the transistor without application and after applying the gate bias of the FET transducer. Characterization of the sensor involves setting the electrical background of the freshly dropped and dried membrane. The standard solution is dropped on the freshly immobilized membrane and repeated drain current measurement is recorded for nearly two hours. The electrical ‘gain’ readings of the device initially show spurious fluctuation both above and below the stable value, eventually to give static readings of the same value. For a particular device, for that particular background electrical medium, this reading of current gain is taken as the electrical baseline. Once the device is subjected to siphoning in the standard solution used, the electrical baseline value remains static; it does not change and can be restored to the equilibrium value by elution.

Once the standard solution serves its purpose of stabilization of the device it washed away and successive droplets of solution of target ion concentration ranging from very low concentration to relatively high concentration prepared by spiking the standard buffer solution with the corresponding salt of metal ion is used to observe the trend or variation followed by the gain of the device with increasing values of target ion concentration. For both target ions tested, the detection limit according to IUPAC norms of a sensor curve is reported to be 10^−11^ M. After each consecutive concentration of the test solution, the sensor exhibits a marked rise of gain value relative to the previously determined baseline gain. Any presence of the target ion in any liquid medium sample can be detected by a similar rise in the electrical output as compared to the electrical baseline of the corresponding buffer. The receptor–ligand binding site modeled interaction of the target-specific binding site in the ionophore and target ion is held responsible for this variation in drain current in correspondence with the concentration of dropped test solution. This binding interaction at the sensing element or ISM coated extended gate electrode unit modulates the applied gate voltage in accordance to the extent of binding of receptors with the target which ultimately models the MOSFET drain current to be a function of the concentration of test solution dropped.

**Figure 3:**
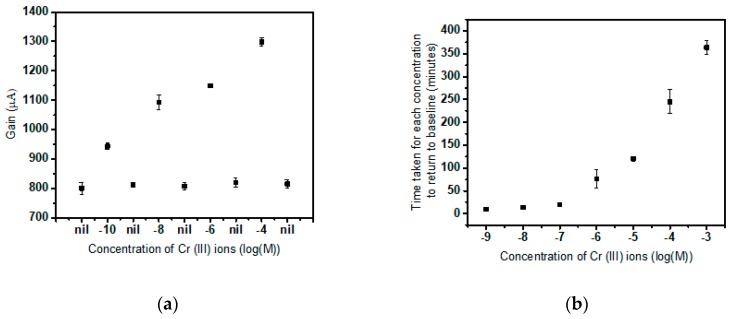
(**a**) The fundamental change in gain and successive return to baseline of the gain values when a stabilized sensor is exposed to consecutive concentrations of the target ion and alternatively eluted by buffer; (**b**) The time taken for different concentrations of target to accomplish elution.

It is observed that once this increase is measured by the sensor, by washing the droplet away and exposing the tested membrane to the standard solution, the gain value gradually drops back to baseline with time allowing for ion-exchange at the membrane solution interface as depicted in Figure 3a. The time required for return to baseline increases with the concentration of exposure. We can infer that more the concentration of target ion the membrane was exposed to, more time is taken for ‘ion exchange’ for the captured target ions to escape from the binding site of the supramolecular receptor and diffuse into the test solution devoid of target ions. The sensor detection is reversible; the sensor can be returned to baseline by eluting with the standard solution after testing with the target salt solution. The gain returns to the electrical baseline once all the bound target ions diffuse out of the membrane to the droplet of the buffer. This reversible nature of the Receptor–Ligand Site Binding Model is reaffirmed here. Regeneration test: before testing the next concentration of Cr(III) test the 0.02X PBS value and make sure the gain value goes back to the original level. By observing the figure, we found that the increase in gain was due to the prolonged exposure to the test solution, and the sensor can be recovered to the same level after the target ion testing. This ion-exchange theory is supported by the observation that more the concentration of the previously dropped solution, more time is taken for completion of elution or for the return to baseline (Figure 3b). It also demonstrates the reversible nature of the sensor and strengthens the reusability of the device multiple times as the effect of binding can be reversed to the pristine state of the stabilized or siphoned membrane.

### 3.1. Calibration of Cr(III) Specific Sensor

**Figure 4:**
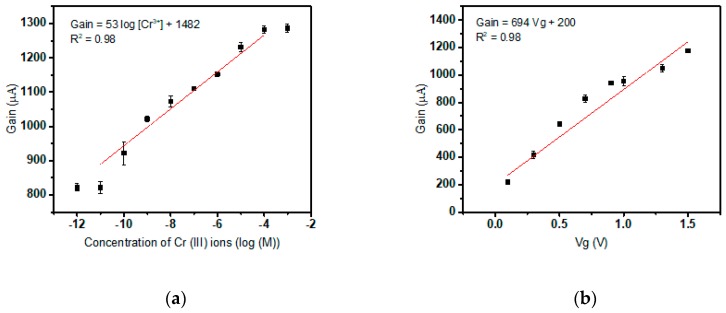

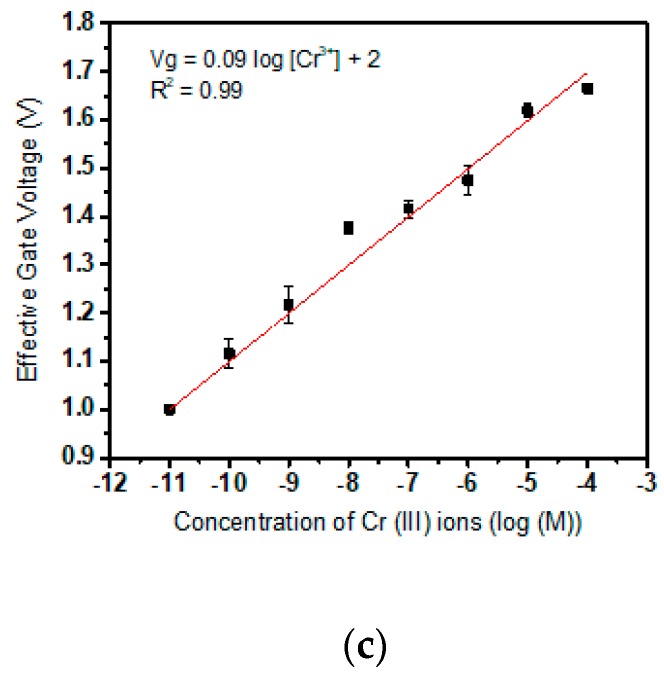
(**a**) Sensor detection curve of Cr(III) target ion; (**b**) Gain versus applied gate voltage in the absence of target ions; (**c**) Graph demonstrating sensitivity of the Cr(III) specific sensor.

For every new fresh device, electrical siphoning or setting of the background is done by stabilizing the current gain values over time. Once this is achieved, each sensor is scrutinized to ensure it responds to a change in gain by testing a droplet with a low concentration of Cr(III) followed by a relatively higher arbitrary concentration a few orders higher. The sensor will show higher gain at a higher concentration which confirms detection, as illustrated in Figure 4a. The sensor is washed back to baseline by dropping the standard solution and repeatedly measuring the electrical sensor output till all the target ions complexed with the ionophore diffuses out through the membrane pores. Now, for the purpose of calibration, the sensor which is presently at electrical baseline is subjected to consecutive droplets of the test solution with successive concentrations of target ions ranging from low (10^−12^ M) to high (10^−3^ M) concentration which ensures saturation of the sensor curve. Figure 4a exhibits the sensor detection curve. The electrical output remains static till a concentration of 10^−11^ M; the detection limit, after which the sensor provides a steady average slope of 53 μA per decade of concentration till the device saturates at 10^−4^ M concentration of Cr(III) target ion. The empirical relation between the concentration of target ions in the test solution and the value of the current gain is revealed to be directly proportional. In this curve, a highly sensitive Cr(III) sensor with an ultralow detection limit of 10^−11^ M and a dynamic range from 10^−11^ M to 10^−4^ M is demonstrated. These observations are abiding the Nernst Equation for ion-selective electrodes or ISE:

(1) $$E_{m}\;=\;C_{1}\;+\;(0.05916\;\log\;A_{\mathrm{anl}})/z$$

E_m_ represents the voltage developed at the ion-selective membrane in contact with the dropped test solution which is analogous to the membrane potential developed across ISM in ISE. At a definite constant gate voltage V_g0_, an increase in the Cr(III) concentration enforce a change in the effective voltage experienced at MOSFET gate terminal (sensing electrode) due to the corresponding change in the potential built across the membrane E_m_. In the above-stated Equation (1), z stands for valency and A_anl_ for target ion concentration. To empirically determine E_m_ developed due to the interaction of ionophore with the target, a fixed solution test was performed. This procedure involved recording the gain values of the device at multiple values of applied gate voltage (V_g0_) ranging from 0 V to 2.0 V in 0.02X PBS buffer solution devoid of any target ion, namely Cr(III) ions. The electrical readings correspond to the gain solely attributed to the applied voltage in the absence of interaction between ionophore and Cr(III) with only the buffer to affect the inter-electrode capacitance. Gain values recorded are uninfluenced by E_m_ and are a result of V_g0_ only. The gain versus V_g_ curve is illustrated in Figure 4b. By converting the linear region from the gain vs concentration curve ranging from 10^−11^ M to 10^−4^ M, a V_g_ vs concentration curve is obtained as in Figure 4c.

Effective Vg = ((Gain at different concentrations − Baseline)/(slope of ‘Gain vs Vg’)) + 1(2)

Equation (2) is utilized to determine the effective gate voltage at the sensing electrode of the FET. This value will include both the applied gate voltage (1 V) and the voltage caused due to change in concentration of the target ion in the dropped test solution. The plot (Figure 4c) depicts the resulting effective V_g_ corresponding to each Cr(III) ion concentrations. By eliminating the applied gate voltage (1V) from the equation, the E_m_ value caused solely by the Cr(III) target ions can be calculated. The plot of the effective gate voltage and concentration has a slope of 99 mV/decade of concentration, which is the sensitivity of the Cr(III) sensor. It has exceeded the Nernst sensitivity for ion-selective electrodes of z = 3 (17 mV/decade of concentration). The value of sensitivity determined by applying the statistical formula of limit of detection (L.O.D.) using standard deviation (S.D.) is also calculated to be nearly 10^−10^ M, one order greater than the experimental result. In Equation (3), ‘blank’ refers to the initial gain value of the sensor before the linear region of sensor curve characteristics.

L.O.D. = blank + 3 S.D. of blank(3)

Thus empirically, to establish a sensor model, the coefficient of correlation between voltage dropped is positive or they are directly proportional to the target concentration. Furthermore, the potential across the membrane is directly proportional to the logarithm of the concentration of target analyte. Contemporary research has justified experimentally that the gap between the gate sensing electrode pair plays a major role in determining the sensitivity of the device. Decreasing the gap approximately exhibits an exponential increase of sensitivity at constant biasing conditions due to the presence of high-density electric field [25]. The semi-empirical model of the extended gate device is:

(4) $$E_{m}\;=\;k\;*\log\;A_{\mathrm{anl}}$$

where k is modeled as a positive coefficient of proportionality which increases with a decrease in the inter-electrode gap of the sensing area.

### 3.2. Calibration of Cr(VI) Specific Sensor

**Figure 5:**
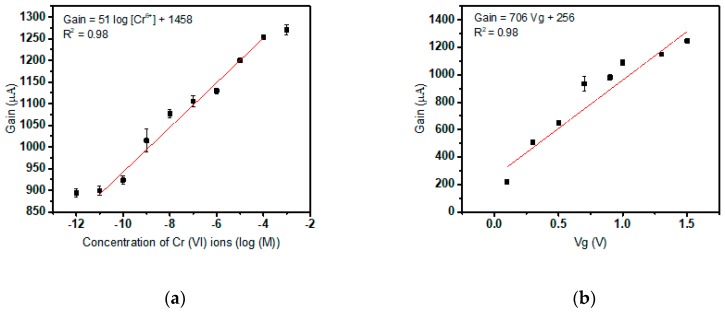

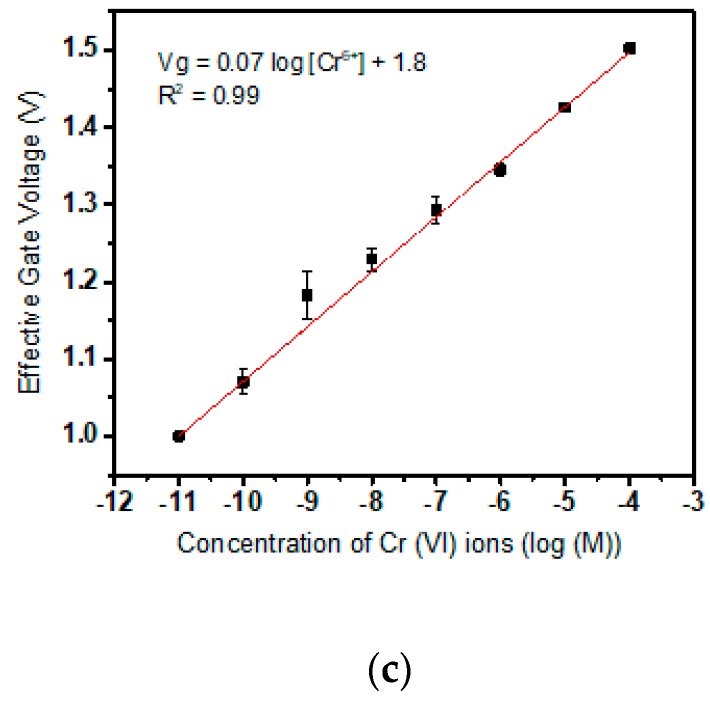
(**a**) Cr(VI) selective sensor gain versus concentration plot; (**b**) Effect of applied gate voltage depicted as gain versus V_g_; (**c**) Sensitivity plot of the Cr(VI) selective biosensor.

The sensors with Quinaldine Red composition drop cast on the sensing element or gate opening is utilized to calibrate and determine the sensitivity of Cr(VI) detection using test solution spiked with increasing concentrations of the target. Initially, the sensors are stabilized against the electrical background of 0.02X PBS within roughly a duration of 2 hours. These sensors are subjected to varying concentrations of Cr(VI) ions and the gain reading is observed. The electrical output of the biosensor system depicts an average increase of 51 mV/ decade of Cr(VI) concentration after the detection limit (10^−11^ M) has been surpassed. The sensor detection curve saturates at nearly 10^−4^ M. Figure 5a depicts the sensor curve having characteristics similar to Cr(III) detection. Now to determine the sensitivity of the sensor, the gain at different gate voltages of the transistor with the test solution devoid of target Cr (IV) ions are plotted (Figure 5b). The applied Vg, 1 V is eliminated the aforementioned two plots are divided to find the gain of the sensor entirely due to E_m_ only or owing to the interaction between Cr(VI) and Quinaldine Red only. Figure 5c is used to calculate the sensitivity of the sensor which is reported to be 71 mV/ decade of concentration. Equation (2) is used similar to Cr(III) sensitivity determination. The detection method can be extended to any liquid medium and the sensitivity offered is much beyond the Nernst limitation of ion-selective electrodes of z= 6 (10 mV/decade of concentration). The limit of detection according to Equation (3) by using standard deviation and slope obtained is 10^−10^ M.

### 3.3. Two-Plate Capacitive Model of The Biosensor

To build an equivalent simplified circuit model of the aforementioned device, the system beyond the terminal of application of V_g0_ can be examined as the sensing element and transducer unit connected in series. The two electrode ISM coated gate sensing element which is responsible for the sensitivity characteristics can be modeled as a sub-system of a two-plate capacitor in series with an equivalent resistor. The ISM composition dropped betwixt the two gold electrodes separated by a short gap comprises the dielectric between the metal plates. The membrane interaction with the target hypothetically will contribute a resistance for the sensing unit.

Contemporary established methods of detection such as ISE operate at saturation region with the reference electrode and sensing area or channel being separated by a considerable distance constituted by the bulk of the solution under test. A synonymous condition is that of electrophoresis where the least value of the electric field must be 1 V/cm [26,27,28]. In the extended gate design, a gap of width 185 µm and applied gate voltage of 1V results in a fringing high field region of approximately 54 V/cm which enhances the sensitivity and facilitates achievement of sensitivity beyond the Nernst limit [25]. This brings the device to high field area of operation as the electric field exceeds the critical value of 1 V/cm so the sensor output is modulated by or highly sensitive to the electric field variations at the sensing area; the membrane and solution interface in particular. The applied gate voltage at the reference electrode drops initially across the membrane and the across the gate dielectric. The entire FET is considered as the sensor entity with performance characteristics that can be replaced by various amplifying transducer models. Hence, the actual signal output to be analyzed is the drop across the FET module.

**Figure 6:**
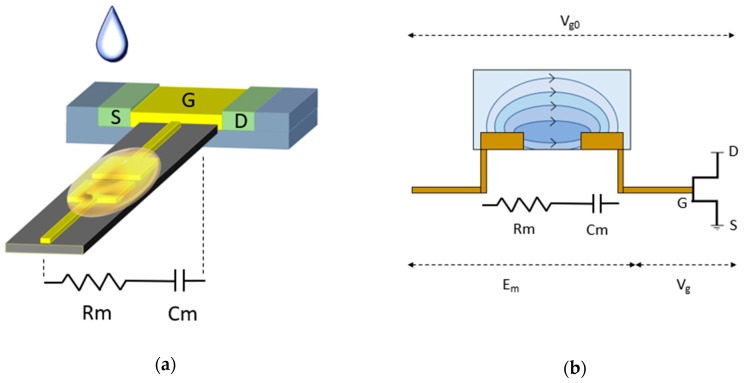
(**a**) Simplified diagram of ‘RC circuit’ configuration in the gate sensing element; (**b**) Illustrative representation of the sensing element and transducer with voltage dropped across series connection.

The drop across the membrane can be considered as the potential being divided across an R-C unit connected in series and hence the voltage divider rule can be applied (Figure 6a). The drop across the gate dielectric or voltage at sensing electrode, and hence ultimately the transistor drain current is modulated by the drop across the membrane which in turn depends on changes inflicted on the capacitance due to interaction with the test solution. The membrane is constituted of porous PVC matrix, hence encapsulates mobile ions which on the application of a potential difference reaches equilibrium by forming complexes between the target (electron deficient) and receptor (electron rich). The movable Cr ions in the dropped test solution enter the porous membrane by ion-exchange or diffusion and interact with the ionophore embedded in the semi-liquid membrane. This phenomenon occurs predominantly at the solution-membrane interface.

V_g_ = V_g0_ − E_m_ (5)

The interaction between the target Cr ions in the solution and supramolecular receptor in the ISM consequently cause the voltage dropped across the membrane, E_m_ to decrease which in turn increases the voltage dropped across the transducer unit, V_g_ which ultimately yields an increase in the transistor drain current with an increase in the concentration of target. The series voltage drop across the sensitive region and FET unit can be perceived from Figure 6b and Equation (5).

### 3.4. Selectivity in the Presence of Interfering Ions

**Figure 7:**
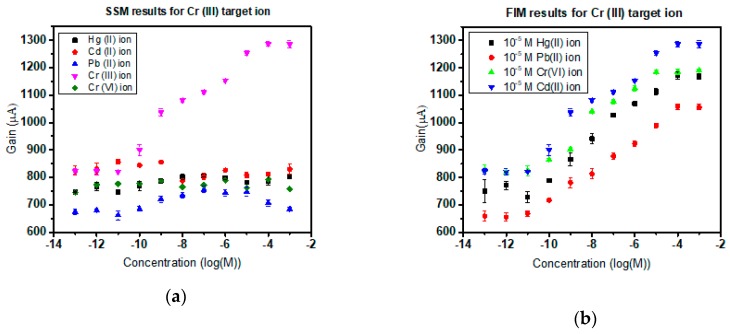
(**a**) Separate solution method of selectivity of Cr(III) ISMFET and its sensor response to other cations; (**b**) Finite Interference method selectivity curve of Cr(III) specific sensor in an environment of interfering cations.

**Figure 8:**
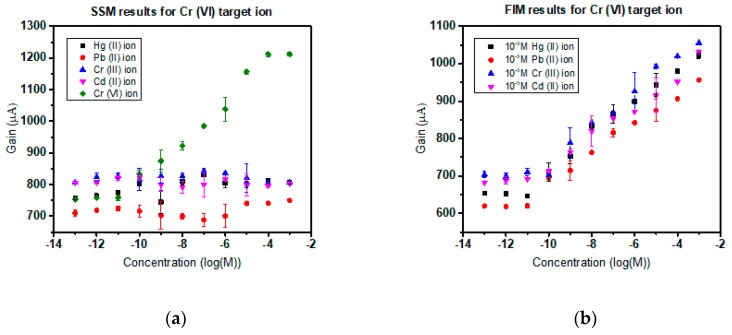

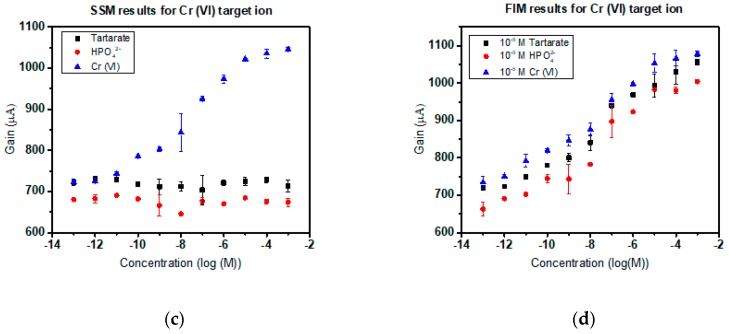
(**a**) Cr(VI) ISMFET SSM selectivity characteristics depicting target-specific interaction of Cr(VI) with ionophore; (**b**) Cr(VI) highly selective FIM characteristics in presence of interfering cations; (**c**) SSM selectivity of Cr(VI) sensor in presence of anions tartrate and phosphate; (**d**) FIM selectivity characteristics of chromate specific sensor in environment of relatively higher concentration of anions tartrate and phosphate.

Subjected to experimental verification, the Cr(III) and Cr(VI) ISMFET have proven to provide commendable selectivity and consistent sensitivity in the presence of common interfering ions. Based on the IUPAC norm of sensor characterization, the heavy metal sensors are scrutinized by the separate solution method (SSM) and finite interference method (FIM). To execute SSM method of selectivity, in Cr(III) ISMFET, 10^−12^ M to 10^−3^ M concentration solutions of Hg (II), Pb (II), Cr(VI), and Cd (II) were prepared and tested to establish the sensor curve. For Cr(VI) ISMFET, the procedure was repeated with 10^−12^ M to 10^−3^ M concentration solutions of Hg(II), Pb(II), Cr(III), and Cd(II). No imminent detection was observed for these cations as sighted in Figure 7a and Figure 8a. This can be attributed to the coordination charge and correspondingly matching cavity size of the supramolecular receptor to ionic radii of target ion [29]. For FIM of Cr(III) ISMFET, the sensor was tested with solutions of 10^−12^ M to 10^−3^ M concentration Cr(III) dissolved in 0.02X PBS containing 10^−5^ M concentration of interfering cations such as Hg(II), Pb(II), Cr(VI), and Cd(II). Similarly, for Cr(III) ISMFET, the procedure is iterated with the buffer containing 10^−5^ M Hg(II), Pb(II), Cr(VI), and Cd(II). Figure 7b and Figure 8b reaffirms that the sensitivity and detection limits of the ISMFET remain unaltered and unaffected by the presence of interfering cations. As Cr(VI) exists as chromate ion, the selectivity was ensured in the presence of interfering anions such as tartrate and phosphate (Figure 8c,d corresponds to SSM and FIM results).

The sensor characteristics were not influenced and were maintained. The other ions do not bind effectively with the receptor to form reversible Receptor–Ligand interaction as they do not match the cavity size or charge. This is a significant advantage over traditional ion-selective membranes where the interfering ions decrease sensitivity and affect limits of detection. This upper hand over contemporary detection methods and short response time make this device an apt choice for implementation in real-time water quality monitoring systems. Also, the application is extensive as any liquid medium, not just water can be tested to detect Cr(III) or toxic Cr(VI) presence in them.

## 4. Conclusions

In this research, we have succeeded in creating a highly sensitive, portable, small size, short response time, easy to fabricate, user-friendly and convenient extended gate chromium ion-selective field effective transistor with multiplexed Cr(III) and Cr(VI) detection. With a lower detection limit of 10^−11^ M for Cr(III) and Cr(VI), we have acquired much better DL than the traditional ISE’s having a detection limit of 10^−8^ M and comparable to the benchtop instruments such as ICP-MS due to the introduction of the novel EDL-FET structure, which is operated in the linear region of the electrical field, and also the extended gate structure which is more convenient to use. Also, the sensor exhibited its ability to operate independently of a specific pH and conductivity range, which allows testing of chromium in other water sources such as groundwater, blood or any fluid food that may.

The corresponding sensor was found to be highly selective toward the target Cr(III) or Cr(VI) ion respectively as compared to other cations. Common interfering ions have no apparent effect on the functioning of this extended gate Cr-ISMFET. This symbolic improvement could be the result of the shortened gap distance between the sensing electrodes. Being much convenient to use and the low production cost attracts our device towards users.

Hence, calibration of this simple novel biosensor can enable the realization of an economical, handy device to measure the concentration of total Cr in water purification systems or even be incorporated into food processing commercial systems or even in medical health-care diagnostics. In conclusion, the sensor developed is a convenient tool to monitor dynamic water quality with a long life and a short response time. The sensor exhibits good sensitivity and detection limit several orders higher than ion-selective membrane for multiplexed Cr(III) and Cr(VI) detection.
